# Targeted therapies for ER+/HER2- metastatic breast cancer

**DOI:** 10.1186/s12916-015-0369-5

**Published:** 2015-06-09

**Authors:** Mutsuko Yamamoto-Ibusuki, Monica Arnedos, Fabrice André

**Affiliations:** Department of Breast and Endocrine Surgery, Graduate School of Medical Sciences, Kumamoto University, Kumamoto, Japan; Department of Medical Oncology, Gustave Roussy Cancer Campus, Villejuif, France; INSERM Unit U981, Gustave Roussy Cancer Campus, Villejuif, France; Department of Medical Oncology and INSERM Unit U981, Gustave Roussy Cancer Campus, 114 Rue Edouard Vaillant, Villejuif, 94800 France

**Keywords:** Breast cancer, Targeted therapy, Cancer genome, Endocrine therapy resistance

## Abstract

The majority of breast cancers present with estrogen receptor (ER)-positive and human epidermal growth factor receptor (HER2)-negative features and might benefit from endocrine therapy. Although endocrine therapy has notably evolved during the last decades, the invariable appearance of endocrine resistance, either primary or secondary, remains an important issue in this type of tumor. The improvement of our understanding of the cancer genome has identified some promising targets that might be responsible or linked to endocrine resistance, including alterations affecting main signaling pathways like PI3K/Akt/mTOR and CCND1/CDK4-6 as well as the identification of new *ESR1* somatic mutations, leading to an array of new targeted therapies that might circumvent or prevent endocrine resistance. In this review, we have summarized the main targeted therapies that are currently being tested in ER+ breast cancer, the rationale behind them, and the new agents and combinational treatments to come.

## Introduction

Endocrine therapy represents a major treatment in all settings of the disease for breast cancers expressing estrogen receptor (ER)-α, which accounts for around 70 % of tumors [1, 2]. During the last two decades, third-generation aromatase inhibitors (AIs), such as anastrozole, letrozole, and exemestane, have become the standard endocrine treatment in postmenopausal women both in advanced and early disease, contributing to an improvement in median survival from 28 to 45 months between the late 1980s and late 1990s [3]. Despite the efficacy of these compounds, response rates for first-line metastatic patients have been described as up to 40 %, with all initial responders eventually developing resistance over time [4]. After progression on an AI, it might still be indicated to pursue with another endocrine agent like fulvestrant, unless there is significant visceral burden and rapid tempo of disease [5]. Other possibilities include treatment with a selective estrogen receptor modulator like tamoxifen or even hormone additive therapies, such as the use of progestins (medroxyprogesterone acetate) [6] and estrogen (ethinyl estradiol) [7, 8].

**Fig. 1:**
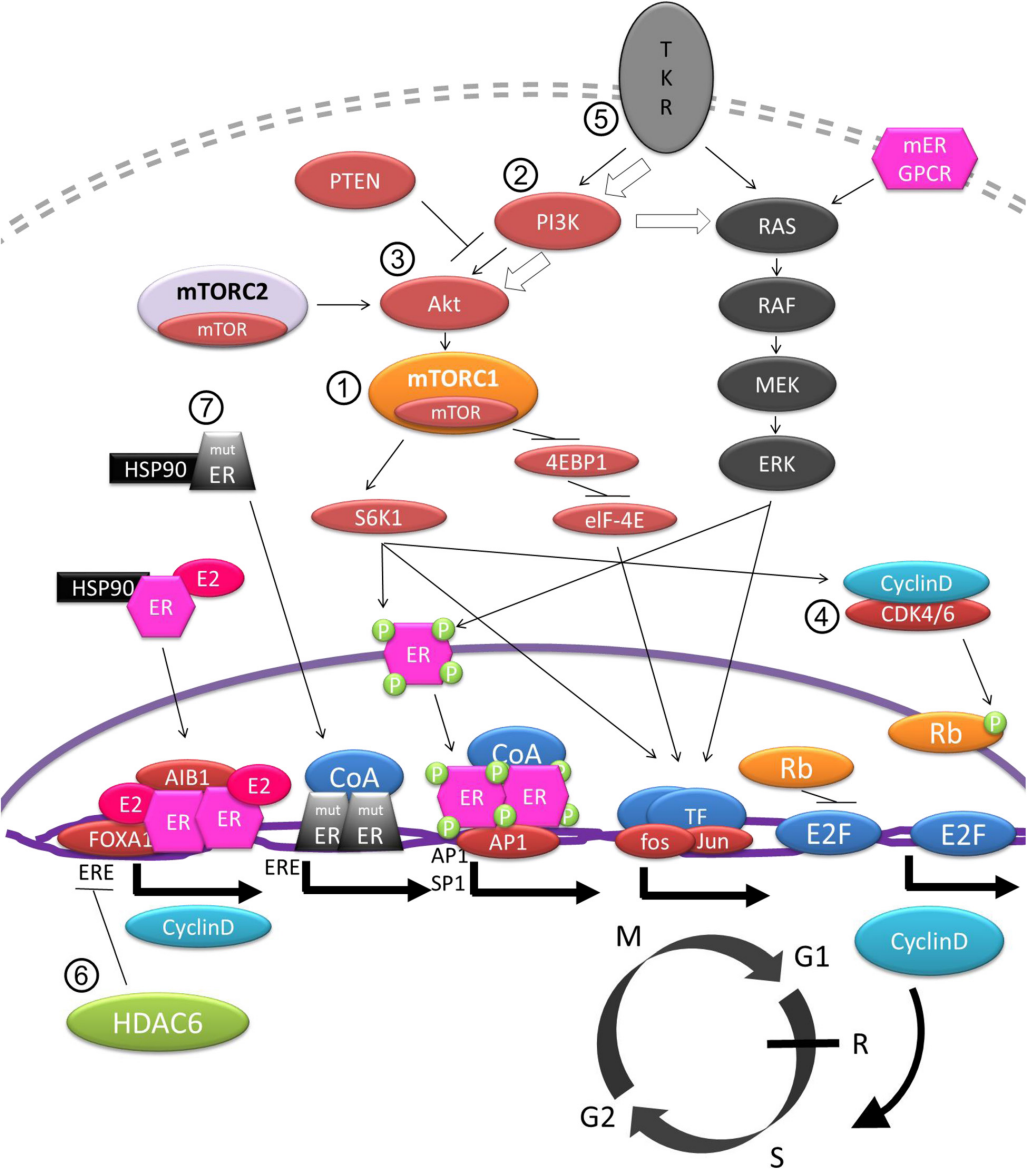
Cross-talk between ER signaling and growth factor signaling pathways described as linked to resistance to endocrine therapy. Classical ER signaling needs to bind to estrogens and HSP90 chaperone protein before binding to transcription start site of target genes such as cyclin D. This transcription activity is partly mediated by histone deacetylation by HDAC6. CyclinD activates E2F transcription via Rb phosphorylation and promotes G1-S transition into the cell cycle for cell proliferation. Suppression of classical ER signaling by endocrine therapy might promote activation of the tyrosine kinase receptor signaling pathways PI3K/Akt/mTOR and RAS-RAF-MAPK via its effectors S6K1 and 4EBP1 to promote ligand-independent activation of ER. Numbers shown in this figure correspond to the function sites of the target agents described in the manuscript. ①mTOR inhibitor: inhibition of mTORC1 down-regulated S6K1 and 4EBP1. In mTOR inhibitor resistance, feedback signaling seems to be activated indicated by white arrow. ②, ③PI3K inhibitors and Akt inhibitors. ④CDK4/6 inhibitors. ⑤FGFR inhibitors. ⑥HDAC6 inhibitors. ⑦Specific inhibitory agents for mutant ER (ex. HSP90 inhibitors). This figure was exclusively drawn for this article

Due to its clinical significance, extensive research has been made in order to determine the potential mechanisms of endocrine resistance. Initial studies had identified the loss of ER expression as responsible for primary resistance, as well as polymorphisms of *CYP2D6* and *CYP19A1* as being responsible for the lack of benefit from tamoxifen and aromatase inhibitors, respectively [9–12], although further studies have not been able to confirm these findings [12, 13]. For both primary as well as secondary resistance, one of the main responsible mechanisms is thought to be the interaction between ER and growth factor receptor signaling via either the phosphatidylinositol-3-kinase (PI3K)/protein kinase B (Akt)/mammalian target of rapamycin (mTOR) pathway, or the mitogen-activated protein kinase (MAPK) pathway which promotes ER phosphorylation (therefore activation) via a non-classical genomic pathway [14] (Fig. 1). More recently, high-throughput technologies studies in ER-positive metastatic breast cancer samples have identified a large number of molecular aberrations in potential driver genes such as *PIK3CA* mutations, *FGFR1* and *CCND1* amplifications (11 %), and *ESR1* mutations (4 %) [12, 15–19], some of them previously linked to endocrine resistance. This, in addition to the recent interest in the cell cycle regulation pathway cyclin D1/cyclin-dependent kinases [20], has resulted in the appearance of several therapies targeting these pathways in order to circumvent or delay the development of endocrine resistance.

In this review, we summarize the rationale and the key clinical data obtained to date with targeting therapies for ER+/human epidermal growth factor receptor (HER)2- advanced breast cancer. This review is complementary to the one reported in the same journal by Migliaccio *et al*. [21], since it will discuss mostly new targeted therapies and mechanisms of resistance.

### mTOR inhibitors

The PI3K (phosphatidylinositol 3-kinase), Akt/PKB (protein kinase B), and mTOR (mammalian target of rapamycin) pathway is an intracellular pathway which mediates gene activation, cell cycle, survival, metabolism motility, and genomic instability [22]. The pathway also contributes to cancer-promoting aspects of the tumor environment, such as angiogenesis [23].

The PI3K pathway is the most frequently altered pathway in breast cancer: the *PIK3CA* gene (encoding the catalytic isoform p110α) is the second most frequently mutated oncogene, and *PTEN* (encoding the phosphatase and tensin homolog) is among the most mutated tumor suppressor genes [24, 25]. In addition, many other molecular alterations within different components of the pathway, including *PIK3CA* amplifications, *AKT1* mutations, and *PTEN* loss, have been observed in ER+ breast cancer [16, 25]. Moreover, the PI3K/Akt/mTOR pathway has been described as potentially intervening in secondary endocrine resistance in ER+ breast cancer [16, 26, 27]. In preclinical models, long-term estrogen-deprived breast cancer cells show an up-regulation of the PI3K pathway leading to a ligand-independent activation of ER by its phosphorylation through the mTOR complex 1 (mTORC1)/S6K1 axis [26, 28]. A series of first-generation mTOR inhibitors have been developed including everolimus (Afinitor, Novartis) [29] and temsirolimus (Torisel, Wyeth) [30] as rapamycin derivatives that inhibit mTOR through allosteric binding to mTORC1. In preclinical models, the use of everolimus in combination with aromatase inhibitors (AIs) results in synergistic inhibition of proliferation and induction of apoptosis [31]. In a randomized, phase II study comparing neoadjuvant everolimus plus letrozole with letrozole alone in patients with newly diagnosed ER-positive breast cancer, the response rate for the combination was higher than that for letrozole alone [32]. Several phase II and III studies including an mTOR inhibitor have been completed in patients with advanced hormone receptor (HR)+ breast cancer, and so far three major randomized trials have reported consistent data in efficacy [33–35] (Table 1). The phase III trial BOLERO (Breast cancer trials of oral everolimus)-2 enrolled 724 patients who were randomized to receive everolimus combined with exemestane (a steroidal AI) versus exemestane plus placebo in postmenopausal patients with HR+ advanced breast cancer previously treated with a non-steroidal AI (letrozole or anastrozole). At the time of the pre-planned analysis, median progression-free survival (PFS) was significantly better for the everolimus plus exemestane arm compared to the control arm (6.9 versus 2.8 months, HR 0.43, 95 % CI 0.35 to 0.54, *P* < 0.001 according to local assessment) [34]. The results of this study led to the approval by the FDA and EMA of everolimus in combination with exemestane in postmenopausal patients with advanced HR+ breast cancer previously exposed to letrozole or anastrozole. Final study results with median 18-month follow-up show that median PFS remained significantly longer with everolimus plus exemestane versus placebo plus exemestane in the overall population [investigator review: 7.8 versus 3.2 months, respectively; HR = 0.45 (95 % CI 0.38 to 0.54); *P* < 0.0001; central review: 11.0 versus 4.1 months, respectively; HR = 0.38 (95 % CI 0.31 to 0.48); *P* < 0.0001] [36]. Updated results have not found a significant benefit for overall survival (OS) with the combination arm, although a trend was observed, with a median OS of 31 months versus 27 months for everolimus versus the placebo arm, respectively; (HR = 0.89; 95 % CI 0.73 to 1.10; *P* = 0.14) [37]. Similarly, the French phase II study TAMRAD (tamoxifen plus everolimus) randomized endocrine therapy alone (in this case tamoxifen) versus tamoxifen plus everolimus in patients again with metastatic ER+ breast cancer previously treated with endocrine therapy [33]. In this trial including a total of 111 patients, clinical benefit rate (CBR) at 6 months (the primary endpoint) was clearly superior for the combination arm as compared with tamoxifen alone (61 % versus 42 % for combined therapy versus tamoxifen alone, respectively (exploratory *P* = 0.045). Time to progression (TTP) was also favorable in the combination arm (8.6 versus 4.5 months; HR 0.54, 95 % CI 0.36 to 0.81, *P* = 0.0021). There was also a benefit in OS for the mTOR-inhibitor arm (not reached versus 32.9 months, HR 0.45, 95 % CI 0.24 to 0.81, *P* = 0.007) [33]. Interestingly, the HORIZON trial, a phase III study in postmenopausal women with HR+ breast cancer that randomized 1,112 patients to receive the mTOR inhibitor temsirolimus in combination with letrozole versus letrozole plus placebo as first-line endocrine treatment, was closed prematurely following an intermediate analysis due to futility [35]. The analysis showed no difference in PFS, the primary endpoint, between the two arms (median PFS of 9 months; HR 0.90, 95 % CI 0.76 to 1.07, *P* = 0.25). There are several ongoing major randomized trials with everolimus in HR-positive advanced breast cancer including BOLERO-4, which will evaluate the benefit from the combination of everolimus and letrozole as first-line treatment (NCT01698918) and might be able to determine if the lack of benefit observed with temsirolimus in the HORIZON study was related to patient population, as preclinical studies have observed that the PI3K/Akt/mTOR pathway is mostly activated after previous endocrine therapy exposure. Finally, the BOLERO-6 trial is an ongoing three-arm phase II randomized study comparing everolimus plus exemestane, exemestane alone, and capecitabine (NCT01783444) in postmenopausal patients with HR+ breast cancer already exposed to endocrine therapy.

**Table 1:** Main clinical trials with targeted agents for ER+/HER2- advanced/metastatic breast cancer: mTOR inhibitors, PI3K inhibitors, and Akt inhibitors

Target	Function	Agent	Trial	Phase	Design		Results with significance/Study status			
					Line	Arm	CBR	TTP	Median PFS	Median OS
mTOR	mTOR inhibitor	Evelorimus	BOLERO-2 [17–19]	III	nsAI failure	EXE + evelorimus vs. EXE			10.6 mo vs. 4.1 mo	n.s.
			BOLERO-4 NCT01698918	II	First line	LET + evelorimus vs. LET	Ongoing			
			BOLERO-6 NCT01783444	II	nsAI failure	EXE + evelorimus vs. EXE vs. capecitabine	Ongoing			
			TAMRAD [20]	II	First line	TAM + evelorimus vs. TAM	61 % vs. 42 %	8.5mo vs. 4.5mo		Not reached vs. 32.9mo
		Tensirolimus	HORIZON [21]	III	First line	LET + tensirolimus vs. LET	Terminated		n.s.	
mTORC	mTORC dual TORC1/2 inhibitor	MLN0128	NCT02049957	I/II		MLN0128 + EXE vs. MLN0128 + FUL	Ongoing			
PI3K	Pan-PI3 K inhibitor	BKM120	BELLE-2 NCT01610284	III	AI failure	Fulvestrant + BKM120 vs. fulvestrant	Ongoing			
			BELLE-3 NCT01633060	III	mTOR inhibitor resisetance	Fulvestrant + BKM120 vs. fulvestrant	Ongoing			
			BELLE-4 NCT01572727	III	First line	BKM120 vs. BKM120 + PTX	Terminated		n.s.	
	PI3K-α inhibitor	BYL719	NCT02058381	II	Premenopausal patients	BYL719 + TAM + Gos vs. BKM120 + TAM + Gos vs. TAM + Gos	Ongoing			
	Pan-PI3K inhibitor/dual PI3K/mTOR inhibitor	XL147/XL765	NCT01082068	I/II	nsAI failure	XL147 + LET vs. XL765 + LET	Completed			
	dual PI3K/mTOR inhibitor	GDC0941/GDC0980	NCT01437566	II	AI failure	Fulvestrant + GDC0941 vs. fulvestrant + GDC0980 vs. fulvestrant	ORR 7.9 % vs. unknown vs. 6.3 %		6.6mo vs. unknown vs. 5.1mo	
Akt	Akt inhibitor	AZD2014	MANTA NCT02216786	II	AI failure	AZD2014 + FUL vs. everolimus + FUL vs. FUL	Ongoing			
		AZD5363	BEECH NCT01625286	I/II	Part B: *PI3CA* mut	AZD5363 + wPTX vs. wPTX	Ongoing			
		MK2206	NCT01277757	II	*PIK3CA* mut *AKT* mut *PTEN* loss/mut	Monotherapy	Terminated			

*Abbreviations*: mTOR: mammalian target of rapamycin, AI: aromatase inhibitor, nsAI: non-steroidal AI, EXE: exemestane, LET: letrozole, TAM: tamoxifen, TORC: mTOR complex, FUL: fulvestrant, PI3K: phosphatidylinositol-3-kinase, PTX: paclitaxel, Gos: goserelin, Akt: protein kinase B

Many efforts have been performed in order to identify potential biomarkers of benefit from mTOR inhibition in patients with breast cancer. Immunohistochemistry (IHC) studies conducted on 55 formalin-fixed paraffin-embedded primary samples from the TAMRAD trial suggested that everolimus is more effective for tumors presenting with high levels of p4EBP1 (a downstream effector of the mTOR pathway), suggesting that baseline mTOR activation might be associated with sensitivity to mTOR inhibition [38]. In parallel, next-generation sequencing studies performed in 309 samples from the BOLERO-2 trial found that the presence of more than one molecular alteration (from four key pathways including *FGFR1/2* amplification, *PIK3CA* mutation, *PTEN* loss, or *CCDN1* amplification) was associated with a lack of benefit from everolimus treatment (HR = 0.78; 95 % CI 0.39-1.54) [17]. These findings suggest that primary resistance to mTOR inhibition might depend on the coexistence of mutations or amplifications in other pathways; therefore, combination therapy with other target agents should be considered for this population. Interestingly, the presence of a *PIK3CA* mutation was not predictive of benefit from everolimus treatment.

### PI3K inhibitor/Akt inhibitor

As mentioned before, PI3K pathway alterations occur in about 70 % of breast cancers and include mutations and/or amplifications of the genes encoding the PI3K catalytic subunits, p110α (*PIK3CA*) and p110β (*PIK3CB*), the PI3K regulatory subunit p85α (*PIK3R1*), and the PI3K effectors *AKT1*, *AKT2*, and *PDK1*. The loss of lipid phosphatases such as *PTEN* can also activate the pathway [17, 39–42]. Preclinically, activation of RTK signaling has been seen to induce transcription of growth-related genes and cause decreases in ER levels and activity, leading to an inferior response to endocrine therapy [43]. Cotargeting this pathway with ER and PI3K inhibitors therefore appears to be a promising therapeutic opportunity for patients with ER+ breast cancer.

The development of PI3K inhibitors is rapidly evolving with newer and more potent compounds entering clinical trials including pan-PI3K inhibitors targeting all isoforms of PI3K, as well as the isoform-specific inhibitors, like inhibitors of the PI3K catalytic subunit p110α, which offer the potential of achieving greater selective target blockade while minimizing off-target effects due to inhibition of other isoforms. Some of the pan-PI3K inhibitors include XL147 [44] and GDC-0941 [45], although the most advanced in clinical research in HR-positive breast cancer is the pan-PI3K inhibitor BKM120 (buparlisib) [46] (Table 1). So far, single-agent clinical trials with pan-PI3K inhibitors have shown modest effect [44, 45, 47]. BKM120 has been evaluated for safety, tolerability, and preliminary activity in combination with letrozole in ER+/HER2- metastatic breast cancer patients refractory to endocrine therapy [48]. The CBR, its primary objective, was of 31 out of 51 patients. Buparlisib's maximum-tolerated dose (MTD) was 100 mg/d. Common drug-related adverse events included ≤ grade 2 hyperglycemia, nausea, fatigue, transaminitis, and mood disorders. Buparlisib is currently being tested in two phase III clinical trials in combination with fulvestrant for patients previously treated with an AI (BELLE-2, NCT01610284) and after resistance of mTOR inhibitor (BELLE-3, NCT01633060). Of note, another phase II/III trial evaluating the benefit of paclitaxel in combination with BKM120 or placebo (BELLE-4, NCT01572727) in first-line advanced HER2-negative breast cancer was recently terminated after an interim analysis due to futility. Another phase II trial of GDC-0941 in combination with fulvestrant (NCT01437566), both in HR+ postmenopausal breast cancer patients, was updated with a result of no PFS significance in the combination group (HR = 0.74; 95 % CI 0.51-1.05), otherwise effective in the ER and PR positive subgroup (HR = 0.44; 95 % CI 0.28-0.69). The combination group showed no correlation in the subgroup with *PIK3CA* mutation, but the patients with *PIK3CA* mutation showed an accurately higher objective response rate (15.8 % versus 3.1 %). Other clinical trials, including the phase II study of XL147 in combination with letrozole (NCT01082068), are currently ongoing.

Preliminary reports about BYL719, a PI3K-α inhibitor, have shown promising activity in patients with heavily pretreated *PIK3CA* mutant breast cancer in a phase I study. Out of the 17 patients treated, 8 (47 %) presented a tumor shrinkage of >20 % [49]. BYL719 is currently being tested in several phase I clinical trials in different types of combinations including with letrozole in postmenopausal patients harboring advanced breast cancer (NCT01791478), with either letrozole or exemestane for the same population (NCT01870505), or in endocrine-sensitive premenopausal HR+ cancer with combined endocrine therapy of tamoxifen and goserelin (NCT02058381). Whether selective *PIK3CA* isoform inhibitors may be superior to pan-PI3K inhibition in safety and efficacy, and which patient populations may benefit the most from their use, are questions yet to be addressed.

In addition, the presence of a negative feedback loop in the PI3K/Akt/mTOR pathway has been demonstrated, in which activation of mTORC1/S6K1 inhibits growth factor signaling to PI3K, exerting negative feedback to restrict insulin and IGF-1 signaling. Loss of this negative feedback mechanism has been shown to occur in cells and tumors exposed to mTOR inhibitors, preferentially those that inhibit mTORC1, which leads to mTORC2 assembly and an increase in phosphorylation of Akt Ser473 [50]. mTOR inhibition also leads to an escape signaling to RAS/RAF/MEK (MAPK signaling) [50, 51] and to an up-regulation of platelet-derived growth factor receptor (PDGFR) signaling [51, 52]. Thus, inhibition upstream to mTOR in the PI3K-Akt pathway might be expected to enhance mTOR inhibition and to exert an anti-tumor effect [17, 39–46, 48, 49, 53].

In order to compensate this Akt activation by this feedback loop caused by mTORC1 inactivation, several different approaches are currently being studied. The first one includes the dual blockade of PI3K and mTOR by the combination of a PI3K inhibitor and an mTOR inhibitor as is currently being tested in a phase II trial of BYL719 in combination with everolimus and exemestane (NCT02077933). Several dual PI3K/mTOR inhibitors are also currently being investigated in phase II studies in different types of tumors including HR+ advanced breast cancer. A phase II randomized trial testing GDC-0941 in combination with fulvestrant (NCT01437566) in HR+ postmenopausal breast cancer patients did not report a significant benefit on PFS (HR = 0.74; 95 % CI 0.51-1.05) [54]. *PIK3CA* mutations were not predictive for the efficacy of GDC-0941. Another phase II trial is ongoing with XL765 in combination with letrozole (NCT01082068). Another approach is the use of mTORC1/mTORC2 complex inhibitors like the four-arm phase II study with AZD2014 in two different schedules (continuous or intermittent) in association with fulvestrant versus fulvestrant + everolimus versus fulvestrant alone as the control arm (NCT02216786).

Of note, several Akt inhibitors are currently being tested in clinical trials to determine their potential benefit, some of them including patients with advanced breast cancer (Table 1), although the trials are still at early stages.

### CDK inhibitor

The cyclin D1 and cyclin-dependent kinase 4 and 6 (CDK4/6) complex pathway is involved in cell cycle regulation and several downstream signals. During cell cycle progression, the cyclin D1-CDK4/6 complex mediates the phosphorylation and inactivation of the retinoblastoma protein (pRb), allowing for cells to progress from the G1 phase to the S phase [55]. In ER-positive breast cancer, the presence of cyclin D1 amplification has been observed, which causes cell cycle deregulation and results in over-proliferation of cancer cells [56]. Therefore, inhibition of the cyclin D1-CDK4/6 complex and the role it might play in restoring cell cycle control in breast cancer is a critical area of study. Results from early *in vitro* and *in vivo* studies have demonstrated that treatment with PD 0332991, a selective cyclin D kinase 4/6 inhibitor, preferentially inhibits proliferation of luminal ER-positive human breast cancer cell lines *in vitro* [57]. Three different oral small-molecule CDK4/6 inhibitors are currently being investigated: palbociclib (Ibrance, Pfizer), abemaciclib (LY2835219, Lilly), and LEE011 (Novartis) (Table 2).

**Table 2:** Main trials of targeted agents for ER+/HER2- advanced/metastatic breast cancer: CDK inhibitors, FGFR inhibitors, HDAC inhibitors, and combined therapy

Target	Function	Agent	Trial	Phase	Design		Results with significance/Study status			
					Population	Arms	CBR	TTP	Median PFS	Median OS
CDK	CDK4/6 inhibitor	Palbociclib	PALOMA-1 [28]	II	First line	Palbociclib + LET vs. LET				
			PALOMA-2 NCT01740427	III	HTfailure	Palbociclib + FUL vs. FUL	Ongoing			
			PALOMA-3 NCT01942135	III	First line	Palbociclib + LET vs. LET	Ongoing			
			PEARL NCT02028507	III	AI failure	Palbociclib + EXE vs. capecitabine	Ongoing			
		LEE011	MONALEESA-2 NCT01958021	III	First line	LEE011 + LET vs. LET	Ongoing			
			MONALEESA-7 NCT02278120	III	Pre/peri menopausal First line	LEE011 + nsAI/TAM + gos vs. nsAI/TAM + gos				
			NCT01709370	II	AI failure	Monotherapy	Ongoing			
		LY2835219	Monarch3 NCT02246621	III	First line	LY2835219+nsAI vs. nsAI	Ongoing			
FGFR	TKI inhibitor FGFR VEGFR PDGFR	Lucitanib	FINESSE NCT02053636	II	1 line HT failure	Monotherapy	Ongoing			
		Dovitinib	NCT00958971	II	With or without FGFR amplification	Monotherapy	21.1 % vs. 12.0 %			
			NCT01528345	II	HT failure	Dovitinib + FUL vs. FUL	Ongoing			
	FGFR1-3	AZD4547	NCT01791985	I/II	1 line nsAI failure	AZD4547 vs. EXE	Ongoing			
HDAC	HDAC inhibitor	Entinostat	ENCORE 301 [35]	II	nsAI failure	Entinostat + EXE vs. EXE	28.1 % vs. 25.8 %		4.3 mo vs. 2.3 mo	28.1 mo vs. 19.8 mo
			NCT02115282	III	nsAI failure	Entinostat + EXE vs. EXE	Ongoing			
			NCT02115594	II	HT failure	Entinostat + FUL vs. FUL	Ongoing			
		Vorinostat	[34]	II	HT failure	Vorinostat + TAM	40 %			
			NCT00616967	II	HT failure	Vorinostat + AI	Ongoing			
Combined	CDK inhibitor/mTOR inhibitor	LEE011 vs. everolimus	NCT01857193	I/II	First line	LEE011 + everolimus + EXE vs. LEE011 + EXE vs. everolimus + EXE	Ongoing			
	Pan-PI3K inhibitor/CDK inhibitor	BYL719 vs. LEE011	NCT01872260	I/II	HT failure	LEE011 + LET vs. BYL719 + LET vs. LEE011 + BYL719 + LET	Ongoing			
	Pan-PI3K inhibitor/PI3K-α inhibitor	BKM120 vs. BYL719 with LEE001	NCT02088684	I/II	HT failure no more than 2 lines of CT	BKM120 + LEE001 + FUL vs. BYL719 + LEE001 + FUL vs. LEE001 + FUL	Ongoing			

*Abbreviations*; CDK: cyclin-dependent kinase, LET: letrozole, FUL: fulvestrant, HT: hormonal therapy, EXE: exemestane, AI: aromatase inhibitor, nsAI: non-steroidal AI, FGFR: fibroblast growth factor receptor, TK: tyrosine kinase, VEGFR: vascular endothelial growth factor receptor, PDGFR: platelet-derived growth factor receptor, HDAC: histone deacetylase, TAM: tamoxifen, PI3K: phosphatidylinositol-3-kinase, CT: chemotherapy

The phase II clinical trial PALOMA-1/TRIO-18 (NCT00721409), testing the efficacy of letrozole with or without palbociclib, was conducted as first-line treatment in HR+ postmenopausal breast cancer patients. Final results have showed a median PFS of 10.2 months (95 % CI 5.7-12.6) for patients in the letrozole alone group, compared with 20.2 months (95 % CI 13.8-27.5) for those given palbociclib plus letrozole (HR = 0.488, 95 % CI 0.319 to 0.748; one-sided *P* = 0.0004) [58]. Notably, the benefit of palbociclib was not outweighed by excess toxic effects, with neutropenia (without an increase in febrile neutropenia) being the most common grade 3-4 adverse event. Several other adverse events were seen in more than 20 % of patients, with increases noted in the palbociclib group, but most were mild or manageable. These results have led to approval of palbociclib in early 2015 by the Food and Drug Administration (FDA) for the treatment of postmenopausal women with ER-positive, HER2-negative advanced breast cancer as initial endocrine-based therapy for their metastatic disease. Palbociclib is also currently being tested in different phase III clinical trials in patients with HR+ postmenopausal advanced breast cancer with different combinations including palbociclib plus letrozole versus letrozole monotherapy in first-line therapy (PALOMA-2, NCT01740427), palbociclib plus fulvestrant versus fulvestrant monotherapy (PALOMA-3, NCT01942135), and palbociclib plus exemestane versus capecitabine (PEARL, NCT02028507), these latter two studies in patients with resistance to AI. Another CDK4/6 inhibitor, LEE011, is currently being investigated in a phase III clinical trial in association with fulvestrant in first-line HR-positive advanced breast cancer (MONALEESA-2: NCT01958021) for postmenopausal patients, and in association with nsAI/TAM plus goserelin for premenopausal breast cancer (MONALEESA-7: NCT02278120). Similarly, abemaciclib is currently being tested in a phase III clinical trial in combination with non-steroidal aromatase inhibitors (letrozole or anastrazole) in postmenopausal women with breast cancer (MONARCH 3: NCT02246621). Results from a previous phase I trial demonstrated that more than 75 % of patients with HR+ breast cancer experienced either partial response or stable disease after a second-line treatment with abemaciclib [59].

Preclinical studies had shown that increased expression of cyclin D1 and pRb were associated with response *in vitro*, as was decreased expression of p16 (a natural CDK4/6 inhibitor) [57]. Unfortunately, in the phase II PALOMA-1/TRIO-18, patient selection on the basis of cyclin D1 amplification or p16 loss was not associated with an improved outcome from palbociclib treatment [58].

A combinatorial drug screen preclinical study has recently identified that CDK 4/6 inhibition sensitizes cells with acquired and intrinsic resistance to PI3K inhibition on multiple *PIK3CA* mutant cancers with decreased sensitivity to PI3K inhibitors. In fact, the combination of CDK 4/6 and PI3K inhibitors exhibited synergistic activity against *PIK3CA* mutant breast cancer cell lines. The reason behind this is the fact that cancers resistant to PI3K inhibitors present with persistence to cyclin D1 pathway activation as determined by the presence of Rb phosphorylation. *In vivo*, the combination of PI3K and CDK 4/6 inhibitors leads to tumor regression in *PIK3CA* mutant xenografts, overcoming intrinsic and adaptive resistance to PI3K inhibition [60].

Based on these findings, several phase I/II studies are currently ongoing with the combination of LEE011 with fulvestrant and BYL719 or BKM120 (NCT02088684), as well as LEE011, BYL719, and letrozole (NCT01872260) in postmenopausal advanced HR+ breast cancer.

### FGFR inhibitor

Fibroblast growth factor receptors (FGFRs) are a family of transmembrane tyrosine kinase receptors belonging to the fibroblast growth factor (FGF) pathway, that upon activation promote cell proliferation, migration, angiogenesis, and survival in cancer cells by the activation of the Ras-dependent MAPK signaling pathway and PI3K/Akt/mTOR. FGFR1 amplification has been identified in around 10 % of HR+ breast cancers, and it has been associated with a worse prognosis, higher Ki67 expression, and resistance to endocrine therapy [61, 62]. Several other less frequent alterations in the FGF pathway have been identified including FGFR2 amplifications, FGFR3 translocations, and amplifications of different ligands like FGF3 and FGF4 that might potentially activate the pathway [41]. Several FGFR inhibitors are currently being investigated in HR+ advanced breast cancer in order to reverse resistance to endocrine therapy (Table 2). Dovitinib (TKI258) is a first-generation oral tyrosine kinase inhibitor (TKI) which inhibits FGFR1-3, VEGFR, and PDGFR. Preclinical data showed that dovitinib inhibits proliferation in FGFR1- and FGFR2- amplified, but not in FGFR-normal breast cancer cell lines [63]. Treatment with dovitinib as monotherapy was evaluated in a phase II clinical trial in women presenting with advanced HR+ breast cancer [63]. Patients were stratified based on the presence of FGFR1 amplification and/or FGF pathway activation determined by qPCR assay. Overall, unconfirmed response or stable disease for more than 6 months was observed in 5 (25 %) and 1 (3 %) patient(s) with FGFR1-amplified and FGFR1-nonamplified breast cancers, respectively. Interestingly, the response rate was 21 % in patients with activated FGF-pathway breast cancer based on qPCR, compared with a 12 % increase in target lesions in patients who did not present with FGF pathway amplification [63]. Dovitinib is currently being investigated in a randomized, placebo-controlled phase II study in combination with fulvestrant (NCT01528345). Another agent, AZD4547, specifically inhibits FGFR1 to 3 and is currently being investigated in ongoing phase I/II trials in patients with advanced HR+ breast cancer after exposure to non-steroidal aromatase inhibitors (NCT01791985), initially in combination with exemestane and posteriorly, after the results of the BOLERO-2 study, with fulvestrant. Both studies include patients with and without alterations in the FGF pathway to determine if there is a role for FGFR inhibition in endocrine-resistant breast cancer and if potential benefit is limited to the presence of a determinate molecular aberration.

### HDAC inhibitor

Numerous epigenetic mechanisms have increasingly being revealed and relate to regulation of gene expression without changing DNA sequence. One of these mechanisms is modification of histone structure by acetylation which contributes to the dilatation of nucleosomal structure and the gathering of transcript factors followed by induction of transcription. The key enzymes, the histone deacetylases (HDACs), remove acetylation to stop the transcription, playing an important role in regulating gene expression [64, 65]. As alterations in HDACs are found in many human cancers [66–68], histone deacetylase inhibitors (HDACi) have aroused interest as a potential treatment for cancer. The first of these new HDACi, vorinostat (suberoylanilide hydroxamic acid), has received FDA approval as monotherapy for treating patients with cutaneous T-cell lymphoma. Moreover, HDAC inhibition has proven to be synergistic or additive with different anticancer agents, including radiation therapy [66], chemotherapy, and new targeted agents [66, 68–70]. In the case of breast cancer, the epigenetic silencing of ER target genes is crucial to ER-independent growth and has been described as a mechanism of endocrine resistance [71]. Based on that, different HDAC inhibitors are being investigated in combination with endocrine therapy in tumors resistant to endocrine therapy (Table 2). Vorinostat has been assessed in combination with tamoxifen in a non-randomized phase II study in patients previously treated with endocrine therapy [72]. The overall response rate was 19 %, and the clinical benefit rate (defined as stable disease > 24 weeks) was 40 %. Similarly, the results from the randomized double-blind phase II study of exemestane with or without entinostat, a benzamide HDAC inhibitor, enrolled 130 patients with resistance to non-steroidal AI. The PFS was 4.3 versus 2.3 months (HR = 0.73, 95 % CI: 0.50-1.07, *P* = 0.055), and the OS was 28.1 versus 19.8 months (HR = 0.59, 95 % CI: 0.36-0.97) for the combination versus the exemestane alone arm, respectively [73]. There is currently ongoing a phase III trial with the same treatment design for the same population (NCT02115282), as well as a randomized phase II study of fulvestrant with or without entinostat (NCT02115594), a phase II trial of vorinostat in combination with AI treatment (NCT00616967), and a phase I trial of abexinostat (S78454/PCI-24781), an oral pan-HDAC inhibitor in combination with tamoxifen. The most important dose-limiting toxicity of these compounds is thrombocytopenia, which is constantly observed and might limit drug combinations [74].

### Targeting *ESR1* mutation

Several reports have recently described the appearance of somatic *ESR1* mutations as a potential mechanism of secondary endocrine resistance in HR+ breast cancer. Robinson *et al*. [75, 76] identified *ESR1* mutations in 6 of 11 (55 %) HR+ advanced breast tumors. Further, Toy *et al*. [66] identified somatic *ESR1* mutations in 9 of 36 (25 %) and in 5 of 44 (11%) ER+ metastatic breast cancers obtained from participants in the BOLERO-2 clinical trial whose disease had progressed during treatment with aromatase inhibitors [34]. A more recent report from Jeselsohn *et al*. [77] found that, overall, the frequency of these mutations was 12% (9/76; 95 % CI, 6 % to 21 %) in metastatic tumors, although it increased up to 20 % (5/25; 95 % CI, 7 % to 41 %) in a subgroup of patients who received an average of 7 lines of treatment. Interestingly, sequencing of ER-positive primary tumors did not identify *ESR1* mutations, including some primary tumors obtained before therapy from a subset of cases with known *ESR1* mutation at metastases [25, 76, 77]. Only Toy *et al*. identified *ESR1* mutations in only 3 % of 183 pretreatment tumor biopsies from BOLERO-2 trial participants [76]. Moreover, none of these groups identified any *ESR1* mutations when sequencing ER-negative breast tumors [75–77]. All these results suggest that *ESR1* mutations are rare in newly diagnosed, untreated breast cancers but appear to be frequently acquired during progression to hormone resistance, especially in the context of estrogen deprivation therapy. To support this theory, these mutations seem also to affect the ligand-binding domain (LBD), encoding p.Tyr537Ser and p.Asp538Gly, which strongly promote classical ER signaling of target genes in the absence of ligand, resulting in the synthesis of receptors with ligand-independent activity and could promote resistance to AI treatment. Both Toy *et al*. [76] and Robinson *et al*. [75] showed that mutant ERα protein can still bind antiestrogens such as tamoxifen and fulvestrant, although higher doses of these drugs were required to inhibit this mutant ERα. This raises the possibility that altered dosing or the development of more potent and/or selective ER antagonists might inhibit residual ER activity and thus overcome resistance in the presence of a mutated ERα.

Yu *et al*. [78] recently reported that targeting heat shock protein (HSP) 90, which is the chaperone protein of ER, may be useful to treat Y537S *ESR1-*mutated tumors. The authors showed that mutant *ESR1* tumors are highly dependent on HSP90, and preclinical studies with the HSP90 inhibitor STA9090 demonstrated cytotoxicity alone and in combination with raloxifene and fulvestrant to *ex vivo* cultured circulating breast tumor cells [78]. Interestingly, they also described that the allele frequency of *ESR1* mutation correlated with the sensitivity to HSP90 inhibition. These findings suggest that *ESR1-*mutation targeted therapy will be possibly oriented by genomic portraits from each patient and that there is a need for more potent or specific antagonists of the mutant forms to block ER signaling as next-generation selective ER modulators (SERMs) and selective ER down-regulators (SERDs).

## Conclusion

The mechanism of resistance to endocrine therapy in patients with ER-positive breast cancer remains a major issue. Previous studies had already identified a cross-talk between the ER pathway and growth factors pathways, mostly PI3K/Akt/mTOR and RAS/RAF/MAPK, as a main potential mechanism responsible for endocrine resistance. Moreover, the use of high-throughput technologies has identified several molecular aberrations present in breast tumors including *PIK3CA* mutations, *AKT*, *FGFR1*, and *CCDN1* amplifications, as well as *PTEN* loss that contribute to the activation of these pathways and therefore might propitiate endocrine resistance via non-classical activation of ER. These findings have been made in parallel to the development of targeted therapies against these driver genes, leading to the approval of two new targeted therapies: everolimus and palbociclib against mTOR and CDK4/6, respectively, in combination with hormonotherapy to circumvent endocrine resistance. More recently, the discovery of somatic *ESR1* mutations in tumors previously treated with endocrine therapy has directed attention to a new mechanism of resistance to endocrine deprivation. This, in addition to the results of currently ongoing clinical trials including combinations of different targeted therapies and a more comprehensive knowledge of the main molecular aberrations, will revolutionize the future management of ER-positive breast cancer.

Many challenges still remain though, as we try to identify the subsets of patients most likely to benefit from these novel targeted agents. A strategy for biological markers-driven selection of target agents for each patient and an integrated form for detecting reproducible key molecular alterations which cause endocrine resistance are mandatory for future precision medicine in this subset of breast cancer.

## Abbreviations

AI: Aromatase inhibitor
Akt: Protein kinase B
CBR: Clinical benefit rate
CDK: Cyclin-dependent kinases
CI: Confidential interval
ER: Estrogen receptor
FGFR: Fibroblast growth factor receptor
GPCR: G-protein-coupled receptor
HDAC: Histone deacetylases
HER: Human epidermal growth factor receptor
HR: Hazard ratio
HR: Hormone receptor
HSP: Heat shock protein
IGFR: Insulin-like growth factor receptor
IRS: Insulin receptor substrate
MAPK: Mitogen-activated protein kinases
mTOR: mammalian target of rapamycin
mTORC: mTOR complex 1
nsAI: non-steroidal AI
OS: Overall survival
PFS: Progression-free survival
PGDFR: Platelet-derived growth factor receptor
PgR: Progesterone receptor
PI3K: Phosphatidylinositol-3-kinase
qPCR: quantitative polymerase chain reaction
S6K1: Ribosomal protein S6 kinase beta-1
SERD: Selective ER down-regulator
SERM: Selective estrogen receptor modulator
TTP: Time to progression
VEGFR: Vascular endothelial growth factor receptor
